# Obesity Accelerates Age Defects in Human B Cells and Induces Autoimmunity

**DOI:** 10.20900/immunometab20220010

**Published:** 2022-03-22

**Authors:** Daniela Frasca

**Affiliations:** 1 Department of Microbiology and Immunology, University of Miami Miller School of Medicine, Miami, FL 33136, USA; 2 Sylvester Comprehensive Cancer Center, University of Miami Miller School of Medicine, Miami, FL 33136, USA

**Keywords:** aging, obesity, adipose tissue, B cells, humoral immunity

## Abstract

Aging is associated with systemic inflammation and decreased production of protective antibodies while the production of autoimmune antibodies is increased. Our results have shown that the human obese adipose tissue (AT), which increases in size with aging, contributes to systemic and B cell intrinsic inflammation, reduced protective and increased pathogenic B cell responses leading to increased secretion of autoimmune antibodies.

With this R56 funding, we have been able to investigate the cellular and molecular mechanisms by which the human obese AT induces intrinsic B cell inflammation and dysfunctional B cell responses, stimulates the secretion of autoimmune antibodies, whose specificity has been characterized, and engages different AT cell types in antigen presentation pathways to allow secretion of these autoimmune antibodies. Briefly, immune cells are recruited to the AT by chemokines released by both non-immune (adipocytes) and by resident and infiltrating immune cells. We have identified several mechanisms responsible for the release of “self” antigens, and we have shown that reduced oxygen availability and hypoxia, cell cytotoxicity and DNA damage induce cell death and lead to further release of pro-inflammatory cytokines, “self” protein antigens, cell-free DNA and lipids. We have also identified different antigen presenting cells in the AT, responsible for the activation of pathogenic B cells, class switch and secretion of autoimmune IgG antibodies. The experiments performed have allowed the discovery of novel mechanisms for pathogenic responses and the identification of pathways to target in order to promote better humoral immunity during aging.

## BACKGROUND OF THE PROJECT

Aging is associated with systemic inflammation, known as inflammaging [1]. Inflammaging has profound effects on humoral immunity, decreasing the production of protective antibodies and increasing that of autoimmune antibodies [2,3].

Obesity is also associated with inflammaging and dysfunctional humoral immunity [4–6] as well as with diminished responses to interventions in patients with inflammatory diseases [7–9], which further affect the outcome of infection, with several health consequences at the individual level.

Aging and obesity are high risk factors for the development of chronic diseases and conditions typical of old age, such as autoimmunity, cancer, psoriasis, atherosclerosis, and inflammatory bowel disease [10,11], as well as insulin resistance (IR) and Type-2 Diabetes Mellitus (T2DM) [12–14]. These findings have suggested that aging and obesity may share cellular and molecular pathways and underlying mechanisms. Inflammaging represents the most important link between obesity, aging and age-associated diseases [1,12,15], as it causes IR, which leads to the transition from metabolically normal obesity to metabolic syndrome, through metaflammation, the process in which excess nutrients promote chronic low-grade inflammation, characterized by high levels of circulating lipids, glucose and reactive oxygen species [16]. Other important common mechanisms shared by obesity and aging are the increase in oxidative stress [17,18], changes in microbiota composition [19,20], epigenetic changes [DNA methylation patterns, post-translational modifications of histones, chromatin remodeling and increased production and maturation of RNAs [21–24], and telomere shortening [25].

The increase in the size of the adipose tissue (AT) is characteristic of obesity. The AT has been considered for long time a storage for excessive amounts of nutrients, but recent work has clearly shown that the AT is also an endocrine and immune tissue. Conversion of the AT from an insulin sensitive (IS) to a IR state during obesity involves expansion of adipocyte volume and remodeling of components of the extracellular matrix together with increased secretion of adipokines and pro-inflammatory cytokines and chemokines, which are involved in the recruitment of immune cells to the AT. The absence of an effective remodeling in response to excess of nutrients promotes metaflammation. Our results initially published on the human obese subcutaneous AT have shown that immune cells are recruited to the AT by chemokines released by non-immune (adipocytes) and by both resident and infiltrating immune cells, generating a positive feedback inflammatory loop [26]. Moreover, we have shown that the obese AT generates autoimmune antibodies, released following processes like reduced oxygen availability (leading to hypoxia and reduced mitochondrial respiration), cell cytotoxicity and DNA damage which induce cell death and release of intracellular protein antigens, cell-free DNA and lipids. All these stimulate class switch and the production of autoimmune IgG antibodies which have been described to be pathogenic in mouse studies [27].

With the focus to evaluate if obesity may be considered a good biomarker of accelerated aging of human antibody responses and with this R56 funding, we have been able to further characterize mechanisms by which the AT induces intrinsic B cell inflammation and dysfunctional humoral immunity, increasing the secretion of autoimmune antibodies whose specificity has been characterized. Additionaly, we have been able to identify different antigen presenting cells in the AT, responsible for the activation of pathogenic B cells, class switch and production of autoimmune antibodies.

## PROGRESS OF THE PROJECT

### Specific Aims

*AIM 1*: Investigate if leptin, the hormone secreted by the AT, induces intrinsic B cell inflammation which we have previously shown is negatively associated with B cell function.
*AIM 2*: Measure the secretion of autoimmune antibodies in the AT and determine their specificity.
*AIM 3*: Identify which cell in the SAT is able to present “self” antigens.

### Recruitment

To perform the experiments proposed in Aim 1, we recruited young (30–50 years) and elderly (≥65 years) healthy individuals, both lean (body mass index, BMI < 24.9) or obese (BMI ≥ 30) at the Department of Family Medicine and Community Health at the UM Miller School of Medicine. To perform the experiments proposed in Aims 2 and 3, we recruited obese adult participants (30–55 years of age) undergoing weight reduction surgeries (breast reduction) at the Division of Plastic and Reconstructive Surgery at the UM Miller School of Medicine. All donors were without diseases and conditions affecting the immune system. Individuals with autoimmune diseases, congestive heart failure, cardiovascular disease, chronic renal failure, malignancies, renal or hepatic diseases, infectious disease, trauma or surgery, pregnancy, or under substance and/or alcohol abuse were excluded from the study. All participants signed an informed consent. The study was reviewed and approved by our Institutional Review Board (IRB, protocols #20070481 and #20160542), which reviews all human research conducted under the auspices of the University of Miami.

### Results

#### AIM 1.

Leptin is an adipokine secreted primarily by the adipocytes [28]. Similar to other pro-inflammatory adipokines, leptin is released into the circulation and its plasma levels are positively associated with BMI. Leptin has endocrine and immune functions and is a potent stimulus for the secretion of pro-inflammatory cytokines by monocyte/macrophages [29–31], neutrophils [31], dendritic cells [32], NK cells [33,34], Th1 [35] and Th17 [36] cells, B cells [37]. Leptin secretion increases with aging [38–40] likely because the AT increases in size with aging [41,42]. Increased leptin has been postulated to be at least one of the mechanisms which may be responsible for the reduced production of protective antibodies in elderly individuals and in individuals with obesity in response to vaccination [4,5].

To identify and characterize cellular and molecular mechanisms through which leptin may be a mechanism of B cell aging, we compared phenotype and function of B cells from the following individuals: young lean, (Y_L_) young obese (Y_O_) and elderly lean (E_L_) individuals, as well as of B cells from Y_L_ individuals in vitro treated with leptin. Our published results [43] have clearly shown that leptin in vitro induces intrinsic B cell inflammation, similar to the levels observed in B cells from Y_O_ and E_L_ individuals. B cell intrinsic inflammation was measured by RNA expression of pro-inflammatory cytokines (TNF-α and IL-6), chemokines (IL-8), micro-RNAs (miR-155 and miR-16), TLR4 and p16, a cell cycle regulator associated with immunosenescence. Leptin in vitro also induced pro-inflammatory B cells, reduced class switch and influenza vaccine-specific IgG production, and these measures were again comparable to those in B cells from Y_O_ and E_L_ individuals. These results altogether confirmed our working hypothesis that leptin is at least one mechanism of immunosenescence in human B cells. A causal relationship between leptin stimulation and the generation of inflammatory B cells which are pathogenic and responsible for the secretion of autoimmune antibodies is currently under investigation in our laboratory.

We also tested the effects of Rapamycin (RAPA), the inhibitor of the mammalian Target Of Rapamycin (mTOR), on B cell function in vitro. We tested the effects of RAPA because leptin is known to induce mTOR activation in both T cells [35,44] and macrophages [45]. Similar to results of clinical studies demonstrating that mTOR inhibition with RAD001, an analog of RAPA, significantly improves influenza vaccine responses in elderly individuals, we provided evidence that RAPA effectively counteracts the leptin-induced down-regulation of class switch in human B cells, at least in vitro. The mechanisms of RAPA in B cells are still not known and deserve future investigation.

#### AIM 2.

Our initially published results have shown that the human obese AT is a site for the differentiation of pathogenic B cells and the secretion of autoimmune antibodies [26]. Although autoantibodies can be directed against a variety of molecules, such as nucleic acids, lipids, and proteins [46], we initially tested the specificity for adipocyte (AD)-derived proteins, released in large amounts after cell death in the calorie-stressed, hypoxic AT. Secretion does not require exogenous stimulation, because the chronic release of “self” antigens in the tissue, due to hypoxia and cell death, stimulate class switch and antibody secretion. This has suggested the importance of understanding antibody specificities.

Therefore, to better characterize these specificities, we performed immunoprecipitation, to enrich culture supernatants in IgG antibodies, and mass spectrometry experiments [47]. Results have shown that the antigens are almost exclusively intracellular or cell-associated antigens, usually not recognized as “self” antigens. In the context of an inflammatory environment like the obese AT, these proteins stimulate the production of autoimmune IgG antibodies which have been described to be pathogenic. These specificities are only present in the plasma of obese but not lean adult individuals [48], as well as in the plasma of elderly individuals as we have recently shown [49]. In support of the above results, previously published work has also shown that the plasma of obese individuals that are IR contains autoantibodies specific for intracellular proteins previously not known as autoantigens [27], suggesting that chronic release of “self” antigens occurs in the AT under obesity conditions.

The specificities that we found present in all individuals evaluated will be used in protein arrays to screen plasma from individuals with other inflammatory conditions and diseases, mainly patients with T2DM and metabolic syndrome, as the majority of these patients are overweight or obese. We are also interested in screening plasma from patients with autoimmune diseases, characterized by chronic activation of B cells which are pro-inflammatory and pathogenic. We have already preliminary evidence that the plasma of Rheumatoid Arthritis patients is enriched in AD-specific IgG antibodies, suggesting that the AT may contribute to pathogenicity also in autoimmune patients.

#### AIM 3.

The AT contains different cell types able to perform antigen presentation. These include non-immune (adipocytes) and immune cells (macrophages, dendritic cells and B cells). The primary function of the adipocytes is to store excess of energy. They also secrete a large number of adipokines and pro-inflammatory mediators contributing to local and systemic inflammation. Experiments conducted in mice have indicated that adipocytes express both CD1d [50,51] and MHC class II [52,53] antigen-presenting molecules, suggesting that they may activate pro-inflammatory cell subsets (Th1 CD4^+^ T cells, CD8^+^ T cells and Tγδ cells) to release pro-inflammatory mediators, leading to the establishment and/or up-regulation of IR. Previously published work has also indicated that adipocytes, through CD1d, can activate iNKT cells, leading to the release of pro-inflammatory cytokines (IFN-γ) that support local inflammation and IR [50,51].

The role of adipocytes on the secretion of autoimmune IgG in the AT, and especially in the human AT, was not known. We then investigated if adipocytes, as compared to macrophages, may stimulate IgG antibody secretion in vitro. Our results have shown that both macrophages (through MHC class II and CD86) and adipocytes (through CD1d) present “self” antigens and stimulate autoimmune antibody secretion [47]. To our knowledge this was and still is a novel finding. Although we cannot exclude that adipocytes and B cells can also directly interact through CD1d, leading to B cell differentiation and antibody secretion, this possibility has not been evaluated during this grant funding period. B cells may directly interact with adipocytes and iNKT cells through CD1d. We are currently investigating if different types of T cells (Tαβ versus Tγδ) engage different antigen-presenting cells (macrophages versus adipocytes) for the presentation of different “self” antigens (proteins versus lipids). This will be evaluated in future studies.

## FUTURE DIRECTION OF THE PROJECT

Future direction of the project will be to study individuals undergoing a diet intervention program for intentional weight loss. We have already an IRB approved for these studies and we have assembled a team of nurses, physycians and nutrition experts to start a small non-randomized study in which participants will be engaged in motivational counseling, to enhance their motivation engaging in a program designed to produce a safe rate of weight loss, through diet and physical activity, with an emphasis on diet. They will be advised to modify their eating behaviors and provided educational support. The recommended diet is aligned with the Diabetes Prevention Program lifestyle intervention behavioral weight loss program (https://dppos.bsc.gwu.edu) with the goal of weight loss of 7% over 6 months when visits are weekly over a 6-month period. This schedule has been shown to be effective in increase retention of participants in the program. At follow-up appointments, patients will be asked about their perceptions regarding their progress, self-identified barriers and self-identified solutions. We have already collected preliminary results on a limited number of individuals (*n* = 9) who have participated in consecutive seasons to our influenza vaccine study in which we measured the effects of obesity-associated inflammation on vaccine-specific humoral immunity. Results have shown that weight loss is associated with a significantly improved influenza vaccine response and to a drastically reduced presence of autoimmune (AD-specific) antibodies in plasma, supporting our hypothesis that reduction in body fat is a way to improve protective humoral immunity while decreasing the pathogenic role of B cells. Our future studies will characterize not only B cells but also other major cell types (T follicular helper cells (T_FH_) and monocytes) involved in humoral responses to the infuenza vaccine. We will thoroughly evaluate their transcriptional profile. Because of the importance of immunometabolism in the regulation of immune responses, we will also investigate the metabolic phenotype as well as metabolic requirements of these immune cells, as our previously published findings have indicated that higher is the metabolic phenotype of immune cells involved in humoral immune responses lower is their capacity to generate protective immunity. These experiments will allow the identification of metabolic pathways to be targeted to improve immune function of vaccine non-responders.
